# The Roles of Phytochemicals in Bronchial Asthma

**DOI:** 10.3390/molecules15106810

**Published:** 2010-10-04

**Authors:** Hee Sun Park, So Ri Kim, Ju Ock Kim, Yong Chul Lee

**Affiliations:** 1 Department of Internal Medicine, Chungnam National University Medical School, Daejeon, Korea; 2 Department of Internal Medicine, Research Center for Pulmonary Disorders, Chonbuk National University Medical School, San 2-20 Geumam-dong, Deokjin-gu, Jeonju, Jeonbuk 561-180, Korea

**Keywords:** antioxidants, bronchial asthma, plant

## Abstract

Despite gaps in our knowledge of how phytochemicals interfere with cellular functions, several natural plant products are utilized to prevent or treat a wide range of diseases. Identification of an agent with therapeutic potential requires multiple steps involving *in vitro* studies, efficacy and toxicity studies in animal models, and then human clinical trials. This review provides a brief introduction on natural products that may help to treat and/or prevent bronchial asthma and describes our current understanding of their molecular mechanisms based on various *in vitro*, *in vivo*, and clinical studies. We focus on the anti-inflammatory and anti-vascular actions of the plant products and other roles beyond the anti-oxidative effects.

## Introduction

Phytotherapy, regarded as an “alternative medicine”, is one of the complementary approaches using extracts from natural origin as medicines or health-promoting agents. In recent years, natural products have received great attention for disease prevention due to their various health benefits and noticeable lack of toxicity and side effects [1]. Many dietary plant products such as grains, nuts, cereals, soy, spices, flaxseed oil, fruits, vegetables, medicinal plants, and herbs contain various phytochemical constituents, such as phenolics, carotenoids, alkaloids, nitrogen and organosulfur compounds, and vitamins.

Recent *in vivo* and *in vitro* studies have shown a potential anti-inflammatory role for some of the known natural compounds with antioxidant activity. Combinations of natural antioxidants offer a variety of mechanisms for reducing oxygen metabolites in tissues, altering signaling pathways, and modulating transcription factors, and they might play key roles in reducing reactive oxygen species (ROS)-dependent damage. Numerous bioactive plant compounds, including several polyphenols, have been recently tested for their antiangiogenic potential.

Asthma and chronic obstructive pulmonary disorder (COPD) are chronic inflammatory diseases of the airways characterized by airway hyperresponsiveness and airflow limitation with acute bronchoconstriction, swelling of the airway walls, chronic mucus plug formation, and airway wall remodeling. Increasing evidence has indicated that oxidative stress, one of the causative mechanisms of asthma, is thought to be a central event in inflammatory responses through activation of transcription factors such as nuclear factor-κB (NF-κB) and activator protein-1 (AP-1), resulting in gene expression of proinflammatory mediators [2]. Antioxidants with good bioavailability may thus protect against the direct injurious effects of oxidants but also may fundamentally alter the inflammatory events in the pathogenesis of various airway diseases.

This review will provide recent objective findings on the anti-oxidative, anti-inflammatory, and anti-vascular actions of plant products in asthma and the future perspectives in this field.

## Oxidative Stress in Asthma

The lung is continuously exposed to oxidants, either generated endogenously by metabolic reactions (e.g. from mitochondrial electron transport during respiration or released from phagocytes) or derived from exogenous sources (e.g. air pollutants and cigarette smoke) [3,4,5,6].

There is now substantial evidence that oxidative stress plays a critical role in the pathogenesis of various lung disorders including asthma, COPD, acute lung injury, pulmonary fibrosis, and lung cancer [7,8,9,10,11]. Asthma is a chronic inflammatory disease of the airway characterized by airway eosinophilia, goblet cell hyperplasia with mucus hypersecretion, and hyperresponsiveness to inhaled allergens and nonspecific stimuli [12,13]. In addition, increased oxidative stress is related to severity of asthma, propagation of inflammatory response, and reduction of responsiveness to corticosteroids [14].

Allergen-activated and recruited inflammatory cells such as eosinophils, macrophages, monocytes and neutrophils from asthmatic patients produce more ROS than do those from normal subjects [15,16]. The constitutive airway cells such as epithelial cells are also a potential source of ROS [17]. In addition to cellular sources of ROS, several asthma mediators including lipid mediators, chemokines, adhesion molecules, and eosinophil granule proteins are potential stimuli of ROS production [18,19,20,21,22]. Besides, some environmental factors such as ozone and diesel exhaust particles may cause an extreme increase of ROS generation in the airway [23]. ROS can perturb airway cells thereby exacerbating many of the pathophysiologic features associated with asthma. ROS directly stimulate histamine release from mast cells and mucus secretion from airway epithelial cells [24,25,26]. The increased release of ROS can also directly damage to epithelial cells and cause cell shedding [15]. Studies have demonstrated that ROS lead to endothelial barrier dysfunction with subsequent increase of cell permeability to fluid, macromolecules, and inflammatory cells [7]. Overproduction of ROS or depression of the protective system in cells results in bronchial hyperreactivity, which is characteristic of asthma [27,28,29].

ROS induce contraction of airway smooth muscle, and this effect is enhanced when the epithelium is injured or removed [27]. In fact, H_2_O_2_ has been shown to cause contraction of airway smooth muscle and airway hyperresponsiveness in animal models [30]. Studies with animal models have indicated that ROS contribute to airway hyperresponsiveness by increasing vagal tone due to damage of oxidant-sensitive β-adrenergic receptors as well as decreasing mucociliary clearance [31,32]. Moreover, ROS can decrease numbers and function of epithelial cilia, increase mucus production, alter release of inflammatory mediators, and cause influx of inflammatory cells [7].

Under pathologic conditions, ROS exert a multitude of actions through various signaling pathways involving mitogen-activated protein kinase (MAPK), phosphoinositide 3-kinase (PI3K)/Akt, and protein kinase C [PKC]), thereby activating pro-inflammatory gene transcription factors such as NF-κB, AP-1, and hypoxia-inducible factor (HIF)-1α [30,33,34,35,36,37,38,39,40,41]. ROS are also involved in production of a number of inflammatory mediators, most notably eicosanoids, by activating phospholipase A2 (PLA_2_) [42,43]. ROS even play a role in responses to innate immune stimuli including lipopolysaccharide and viruses as well as to acquire immune responses such as antigen interactions with IgE or IgG antibodies [44,45,46].

## Antioxidant System in the Lung – Balancing Intracellular Oxidation

The lung has several natural antioxidant mechanisms to neutralize overproduced oxidants (ROS, reactive nitrogen species, and lipid peroxides), which include enzymatic as well as non-enzymatic antioxidants. These antioxidant defense systems form a tightly regulated network to resist any change in the redox environment of intra- and extracellular space [47]. Enzymatic antioxidants include catalase, glutathione peroxidase (GPX) and superoxide dismutase (SOD), and non-enzymatic antioxidants are vitamin C, vitamin E, albumin, uric acid, ceruloplasmin, and glutathione (GSH) [48,49,50]. Changes in these enzymatic and non-enzymatic antioxidants can disrupt homeostasis of ROS in bronchial cells.

Deficiency of endogenous antioxidant defenses has been reported in asthma [51]. Devereux and colleagues have proposed that individuals in westernized societies have progressively reduced their consumption of fruit and vegetables and as a result, decreased pulmonary antioxidant defenses, making them more susceptible to inhaled irritants and allergens [52]. As many antioxidants are supplied from the diet, attention has been paid to intake of the micronutrient antioxidants (vitamins A, C, and E, polyphenols and carotenoids) and to know how it may help to protect individuals from an oxidizing environment and/or inflammatory airway disease.

Plants have two primary strategies to detoxify harmful oxidant radicals; one is direct enzymatic breakdown of oxidant radicals using SOD, catalse, ascorbate peroxidase, peroxidase, glutathione reductase and monodehydroascorbate reductase [53]. These enzyme systems convert various oxidant radicals to reduced products. The second strategy is synthesis of antioxidant molecules such as vitamin C and vitamin E. These antioxidant compounds possess a hydroxyl group (-OH) on the ring structure, with an associated electron deficiency, which are highly reactive towards ROS [54,55].

## Antioxidant Molecules in Plants and the Their Role in Bronchial Asthma

In the next paragraphs, we described the antioxidant and anti-inflammatory properties of some important natural bioactive compounds, which exert favorable effects on bronchial asthma (Table 1). These agents would be useful not only for reducing oxidative stress in asthma but also for the control of inflammation. Most of their actions are related to their ability to direct enzymatic breakdown via endogenouse anti-oxidative enzymes, synthesis of various anti-oxidants and quenchers, thereby inhibiting cytokine, chemokine or adhesion molecule synthesis and/or action. However, only a few plant-derived compounds have been submitted to clinical trials to test their potential as antioxidative and anti-inflammatory agents.

**Table 1:** Plant antioxidants and their postulated mechanism of effect.

Bioactive compounds		Activity and Potential mechanisms of effect
**Polyphenols**		
*Flavonoids*	Flavans	Donation of hydrogen atom to radicals
	Flavanones	Chelation of redox-active metals
	Isoflavanones	Inhibition of lipid peroxidation
	Flavones	Regulation of the enzyme activities
	Isoflavones	Inhibition of mast cell/basophil activation
		Inhibition of eosinophlic degranulation
	Anthocyanidins	Switching allergic immune response to Th1 profile
	Chalcones	Regulation of various transcription factors and mediators involving angiogenesis: HIF-1, VEGF, MMPs, EGFR and inhibit NF-κB, PI3K/Akt, and ERK1/2 signaling pathways
	Flavonolignans	
*Curcumin*		Prevention of lipid peroxydation
		Radical scavenger/neutralizer
		Decrease the levels of iNOS
		Regulation of cytokines such as IL-2, IL-5, and GM-CSF through maintaining HDAC2 activity
		Inhibition of mast cell activation
		Inhibition of neutrophil function
*Resveratrol*		Inducing and stabilizing antioxidant enzymes
		Inhibition of prostaglandin production
		Decrease the phosphorylation of ERK1/2, cyclooxygenase-2 activity, and activity of various transcription factors including NF-κB, STAT3, HIF-1α, and β-catenin
		Inhibit protein kinases (src, PI3K, JNK and Akt)
		Inhibit production of inflammatory mediators (IFN-γ, TNF, COX-2, iNOS, CRP and various interleukins)
		Sirtuin1 activation
		Modulate innate immune response
		Inhibition of angiogenetic pathway that is mediated through expression of MMPs, VEGF, cathepsin D, ICAM-1 and E-selectin
**Antioxidant vitamins**		
*Carotenoids*	Lycopene	Quenches singlet oxygen without degradation
	Lutein	Regulation of various transcription factors (AP-1, NF-κB)
	β-cryptoxanthin	Supression production of inflammatory cytokines
	α-carotene	Reduce induction of IGF-1
	β-carotene	
*Vitamin C*	Ascorbic acid	Donation of hydrogen atom to radicals
	Dehydro-ascorbic acid	Inhibiting the JNK/AP-1 signaling pathways
		prostaglandin inhibition
*Vitamin E*	Tocopherols	Hydroperoxide scavenger
	(α, β, γ, and δ)	Modulation of the functional activity of T-lymphocytes and enhance the phagocytic activity of peripheral granulocytes
	Tocotrienols	
	(α, β, γ, and δ)	
		Inhibit monocyte response to LPS and LPS-induced degrdation of IκB and JNK activation
		Regulation of endothelial cell signals
		Membrane stabilization
		Inhibition of IgE production
**Organosulfur compounds**		
*α-lipoic acid*		Quenches reactive oxygen species
		Regenerates/recycles endogenous and exogenous antioxidants
		Chelates redox metals
		Modulate the activity of transcription factors
**Volatile compounds**		
*Phytoncides*		Insecticide
		Antibacterial/antifungal activity
		Radical scavenging activity
		Enhance the activity of NK cells
		Restoring antioxidants
		Modulate the activity of transcription factor, NF-κB
		Attenuate allergic inflammation

AP-1, activator protein-1; COX, cyclooxygenase; CRP, C-reactive protein; EGFR, epidermal growth factor receptor; GM-CSF, granulocyte macrophage colony-stimulating factor; HDAC, histone deacetylase ; HIF, hypoxia inducible factor; IκB, inhibitory kappaB; IFN, interferon; iNOS, inducible nitric oxide synthase; JNK, Jun N-terminal kinase; LPS, lipopolysaccharide; MMPs, matrix metalloproteinases; NF-κB, nuclear factor-kappaB; NK, natural killer; PI3K, phosphoinositide 3-kinase; STAT, signal transducer and activator of transcription; TNF, tumor necrosis factor; VEGF, vascular endothelial growth factor

### Flavonoids

**Figure 1:**
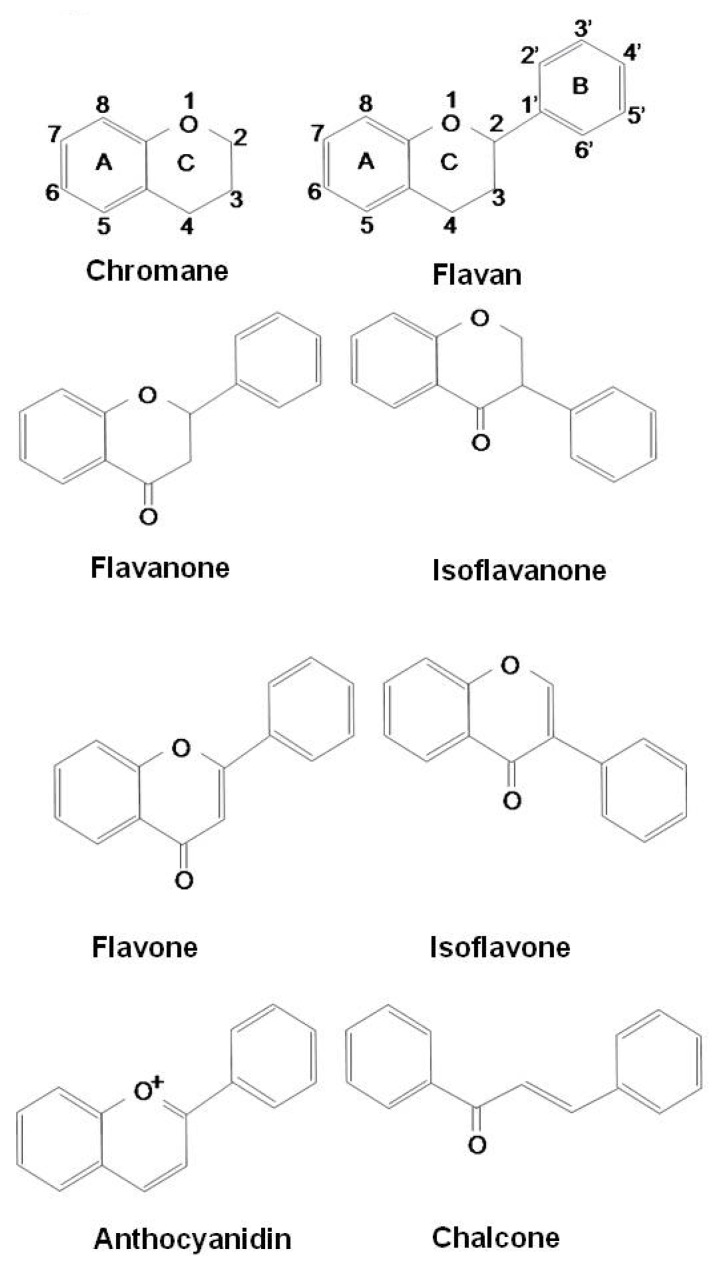
Structures of flavonoids.

Flavonoids constitute the most important single group of polyphenols of low molecular weight polyphenolic secondary plant metabolites, with more than 8,000 compounds described [56,57,58]. They are found in fruits, vegetables, nuts, seeds, stems, flowers, roots, tea, wine, and coffee and are common substances in our daily diet [59,60]. Their structure is a heterocyclic hydrocarbon, chromane, and substitution of its ring C in position 2 or 3 with a phenyl group (B-ring) results in flavans or isoflavans. An oxo-group in position 4 leads to flavanones and isoflavanones. The presence of a double bond between C2 and C3 provides flavones and isoflavones. An additional double bond in-between C1 and C2 makes these compounds colorful anthocyanidins. Based on their structure, flavonoids are categorized into eight groups: flavans, flavanones, isoflavanones, flavones, isoflavones, anthocyanidins, chalcones, and flavonolignans (Figure 1).

Flavonoids interfere with oxidation of lipids and other molecules by rapid donation of hydrogen atoms to ROO• radicals. This hydrogen (electron) donating ability of a flavonoid molecule that acts to scavenge reactive radical species is primarily associated with the presence of a B-ring catechol group (dihydroxylated B-ring) [61]. One important structural feature, which is partially responsible for the antioxidant properties of flavonoids, is the presence of 2,3-unsaturation in conjugation with a 4-oxo group in the C-ring. In addition, the presence of functional groups involving both hydroxyl groups of B-ring and the 5-hydroxy group of A-ring is an important contributor in the ability of flavonoids to chelate redox-active metals and thus prevents catalytic breakdown of H_2_O_2_ [61]. The phenoxy radical intermediates are relatively stable, so they do not initiate further radical reaction and act as terminators of the reaction chains by interacting with other free radicals. Flavonoids are ideal scavengers of peroxyl radicals due to their favourable reduction potentials relative to alkyl peroxyl radicals, and thus they are effective inhibitors of lipid peroxidation [61]. Therefore, this strong anti-oxidative property of flavonoids has made them protective against airway diseases linked to oxidative stress [62,63]. In fact, several epidemiologic data suggest beneficial effects of flavonoids on asthma. A population-based case-control study has suggested that apple consumption or red wine intake were negatively associated with asthma prevalence or severity, respectively, perhaps due to a protective effect of flavonoids [64]. Moreover, a 30-year longitudinal epidemiological study reported that the incidence of asthma is lower in populations with higher intake of flavonoids [65].

Beyond anti-oxidative effects, flavonoids inhibit release of histamine and other preformed granule-associated mediators by inhibiting the activation of basophils and mast cells [66]. Flavonoids also inhibit synthesis of IL-4, IL-13, and CD40 ligand but initiate generation of new phospholipid-derived mediators. One of the well characterized flavonoids, quercetin inhibits eosinophilic secretion of Charcot-Leyden crystal protein and eosinophil cationic protein in a concentration-dependent manner [67]. Moreover, the inhibitory action of quercetin on other inflammatory cells appears to surpass any other clinically available compounds [57]. Very recently, Li *et al*. have also demonstrated that apigenin exhibits an anti-inflammatory activity in a murine asthma model and can switch the immune response to allergens toward the T-helper type 1 cell (Th1) profile [68]. These findings suggest that flavonoids are anti-allergenic and anti-inflammatory agents effective in treating/preventing of asthma.

Vascular changes are one of the major components for asthmatic pathogenesis [69]. The changes include an increase in vascular permeability, vascular dilation/engorgement, and vasculogenesis/angiogenesis [69]. Flavonoids and their related compounds have been shown to modulate expression of HIF-1, VEGF, matrix metalloproteinases (MMPs), and epidermal growth factor receptor but also inhibit NF-κB, PI3K/Akt, and ERK1/2 signaling pathways [70,71,72,73,74,75,76,77]. These observations have suggested that flavonoids as well as their related compounds inhibit certain steps of angiogenesis that are cell migration, microcapillary tube formation, and MMP expression [77,78].

### Curcumin (diferuloylmethane)

The active component of turmeric is curcumin, a polyphenolic phytochemical with anti-inflammatory, antiamyloid, antiseptic, antitumor, antiallergic, and anti-oxidative properties [79]. In addition to its culinary uses, curcumin has been used as a traditional medicine for liver disease (particularly jaundice), indigestion, urinary tract diseases, blood purification, inflamed joints (rheumatoid arthritis), insect bites, dermatological disorders, and atherosclerosis [79]. Curcumin has been shown to be eight times more powerful than vitamin E in preventing lipid peroxidation [79,80]. It has also been suggested that curcumin plays a role in reducing oxidative stress by downregulating nitric oxide (NO) formation, scavenging or neutralizing free radicals, especially superoxide anion, and breaking the oxidative chain reaction caused by free radicals [80,81,82].

Curcumin has been implicated as an anti-inflammatory agent, but the precise mechanisms of its action are largely unknown. However, studies have demonstrated that curcumin decreases the level of inducible NO synthase induced by IFN-γ in lung tissue and expression of cytokines such as IL-2, IL-5, and GM-CSF through acting as a HDAC activator or inhibits histamine release from mast cells [79,80,83,84,85]. Such regulations by curcumin attenuate asthma phenotypes, reducing asthmatic symptoms, recruitment of eosinophils to the airway, and airway hyper-responsiveness. These findings indicate that curcumin may be useful as an adjuvant therapy for asthma.

### Resveratrol

**Figure 2:**
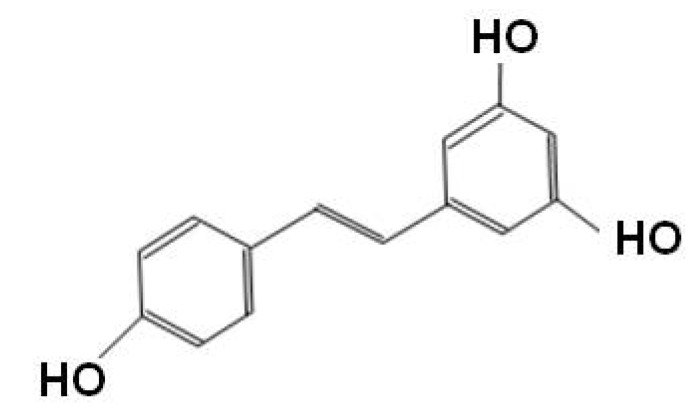
Sturucture of resveratrol.

Resveratrol (3,4΄,5-trihydroxystilbene), more famous for being a constituent of red wine, is a phytoalexin found in various plants including grapes, berries, and peanuts [86]. Resveratrol is one of the polyphenolic compounds consisting of two phenol rings connected by a 2-carbon methylene bridge with several benefits on cardiovascular protection, anticancer effect, and a positive regulator of several aspects of metabolism (Figure 2) [87,88,89]. 

Resveratrol is capable of scavenging intracellular ROS by inducing and stabilizing antioxidant enzymes such as catalase, SOD, and glutathione peroxidase hemoxygenase [90,91]. In addition to its reducing properties, resveratrol has been shown to attenuate inflammation via inhibition of prostaglandin production and to decrease the phosphorylation of ERK1/2, cyclooxygenase-2 activity, and activity of various transcription factors including NF-κB, STAT3, HIF-1α, and β-catenin [92,93,94,95,96,97]. Resveratrol has also been known to inhibit protein kinases (e.g. src, PI3K, JNK, and Akt) and production of inflammatory mediators (e.g. IFN-γ, TNF, COX-2, iNOS, CRP and various interleukins) [98,99,100,101,102,103]. Moreover, recent studies have reported that resveratrol activates sirtuin1 (SIRT1) which is linked with apoptosis and longevity [104,105]. SIRT1 modulates poly (ADP-ribose) polymerase-1 (PARP-1) activity upon DNA damage. Activation of SIRT1 by resveratrol leads to a decrease in PARP-1 activity and promotes cell survival, which can attenuate the inflammatory reaction.

Resveratrol is also able to modulate innate immune response by inhibiting expression of costimulatory molecules (CD80 and CD86) and major histocompatibility complex classes I and II in bone marrow-derived dendritic cells [106] and to inhibit angiogenesis pathway that is mediated through expression of MMPs, VEGF, cathepsin D, ICAM-1, and E-selectin. These findings suggest that resveratrol can be a very attractive compound for preventing/treating asthma since this compound displays multiple therapeutic effects, showing antioxidative, anti-inflammatory, immume modulating, and vascular protective property.

## Antioxidant Vitamins

### Carotenoids

Total vitamin A consists of preformed vitamin A (retinol) and provitamin A compounds, defined as carotenoids, the most important being beta-carotene. Carotenoids are pigments found in plants and microorganisms [107]. Various studies have indicated that carotenoids may prevent or inhibit certain types of cancer, atherosclerosis, immunological disorders, asthma, and other diseases [56,108,109,110,111,112]. The antioxidant activity of carotenoids arises primarily as a consequence of the ability of the conjugated double-bonded structure to delocalize unpaired electrons [113]. Among them, β-carotene quenches singlet oxygen without degradation and reacts with free radicals such as the peroxyl, hydroxyl, and superoxide radicals. This process prevents chain reactions that could result in lipid peroxidation or DNA damage, both of which have been postulated as being precursors of disease processes [56,114,115].

To date, our understanding of the role of carotenoids in asthma is limited. Based on the results from the literature search, carotenoids, in particular, β-carotene seem to exhibit bimodal behavior depending on some factors; they show antioxidant behavior at low oxygen partial pressures, usually below 150 Torr, but at high pressures of oxygen, they may lose antioxidant property, or even become pro-oxidants [116]. Concentration of carotenoids also influences their anti/pro-oxidant properties in a similar manner; at high carotenoid concentrations, there is a propensity for pro-oxidant behavior [116,117]. In asthmatics, reduced levels of total carotenoids (lycopene, lutein, β-cryptoxanthin, α-carotene and β-carotene) in the whole blood were observed, as compared to healthy controls [118]. Additionally, hypovitaminosis A induces respiratory epithelial changes, such as metaplasia, and may predispose to respiratory infections, which may exacerbate acute asthmatic attacks in children [119,120,121,122].

Carotenoids may regulate activation of a variety of transcription factors [123]. Treatment of cells exposed to oxidative stress with β-carotene suppresses oxidative stress-induced activation of NF-κB and production of IL-6, TNF-α, and inflammatory cytokines. Carotenoids may influence the process of apoptosis in healthy cells. While the pro-apoptotic protein Bax is down-regulated after induction of external stimuli, β-carotene is able to increase expression of the anti-apoptotic protein Bcl-2 in normal cells [124]. In addition, β-carotene exhibits a pro-apoptotic effect in colon and leukemic cancer cells, and this effect occurs by a redox-dependent mechanism linked with NF-κB activity [125]. Lycopene has also been shown to regulate transcription factors. Mammary cancer cells treated with lycopene have shown to inhibit AP-1 binding and reduce induction of insulin-like growth factor-I. These dual roles of vitamin A including carotenoids on apoptosis provide the capability of carotenoids as an effective anti-inflammatory agent in various diseases.

### Vitamin C

Vitamin C (ascorbic acid) is a very important and powerful antioxidant, which works in aqueous environment of the body. It presents in two biologically active forms, ascorbic acid and its oxidized derivative, dehydro-ascorbic acid, which are interconvertible [126]. Vitamin C acts as a hydrogen donor to reverse oxidation and protects membranes against oxidation (*i.e.*, reducing agent). However, its effects, at very high doses, have been a subject of intense debate for many years [127,128]. Vitamin C prevents acid-catalyzed generation of *N*-nitroso compounds and competes with the secondary amines and amides for nitrosating species [129]. The intake of high doses of vitamin C (up to 2,000 mg/day) has been tried but its beneficial action has never been really established [130,131]. Some clinical studies have demonstrated that low level of vitamin C is associated with asthma risk in children, and asthmatic subjects had significantly decreased ascorbic acid and conversely supplementation of vitamin C benefits asthmatic adults who smoke, reducing cough and wheeze [132,133].

Vitamin C is also able to regulate factors that may influence gene expression, apoptosis, and other cellular functions. In fact, it protects against cell death triggered by various stimuli, and major proportion of this protection is associated with its antioxidant ability [129]. Vitamin C regulates the AP-1 complex including Fos and Jun superfamilies. Treatment of cells exposed to UV-B irradiation with vitamin C results in a 50% decrease in JNK phosphorylation, which activates AP-1, therewith inhibiting the JNK/AP-1 signaling pathways [134]. At present, however, evidence from randomized-controlled trials is insufficient to recommend a specific role for vitamin C in the treatment of asthma due to variable study design and generally poor reporting system [135].

### Vitamin E

Vitamin E is a fat-soluble vitamin that exists in eight different forms. It is consists of a group of substances belonging to two closely related families, the tocopherols and tocotrienols, with each existing in a number of isomeric forms (α, β, γ, and δ) [136]. Alpha-tocopherol is the most active form of vitamin E in humans and is considered the major membrane-bound antioxidant employed by cells [137,138,139,140]. Its main antioxidant function is protection against lipid peroxidation [141]. Alpha-tocopherol is converted to α-tocopherol radical by donation of labile hydrogen to a lipid or lipid peroxyl radical, thus preventing the increase of oxidative radicals being more produced. In the case of damage to fatty acids (e.g. polyunsaturated fatty acids in cell membranes), lipid peroxidation alters the function of the cell membrane and possibly cause irreversible damage to metabolic pathways [114,115,142,143]. There is an interaction between vitamin E and other nutrients, particularly selenium and vitamin C in the antioxidant role. The α-tocopherol radical can thus be reduced to the original α-tocopherol form by ascorbic acid [144]. 

Normal plasma level of tocopherol may enhance lipoxygenation of arachidonic acid, whereas high tocopherol level exerts a suppressive effect as a hydroperoxide scavenger. Receptor-mediated activation of neutrophils in individuals with asthma results in synthesis of leukotrienes, which are known to play important roles in asthma pathophysiology [126]. Vitamin E may induce immunological effects via modulation of the functional activity of T-lymphocytes and enhance the phagocytic activity of peripheral granulocytes [145].

Several epidemiological trials have reported the efficacy of vitamin E supplements in bronchial asthma [146,147,148,149]. Vitamin E intake was generally unrelated to asthma status but was significantly lower in severe asthma than in mild asthma [133]. Currently, any randomized controlled trial and case-control study do not confirm the positive effect of an eventual supplementation of vitamin E. However, there are opposing regulatory functions of vitamin E isoforms thus we have to be cautious on interpretation of vitamin E studies. γ-Tocopherol also appears to be a more potent anti-inflammatory agent than α-tocopherol [150]. It decreases systemic oxidative stress and inhibits monocyte response to lipopolysaccharide (LPS) and LPS-induced degradation of IκB and JNK activation. There is a contradictory study demonstrating that γ-tocopherol elevates inflammation and ablates the anti-inflammatory benefit of the α-tocopherol by regulation of endothelial cell signals during leukocyte recruitment in experimental asthma [151]. Dietary tocopherols are taken up from the intestine and transported via the lymph to the blood and then to the liver. In the liver, α-tocopherol is transferred to plasma lipoproteins, resulting in retention of γ-tocopherol in tissues at 10% that of α-tocopherol [152]. On interpretating these two contradictory results, we should consider their serum levels with caution since low plasma level of γ-tocopherol (1.2–7.0 μM) may act as pro-oxidant, while higher level of γ-tocopherol (19.5 μM at 8 days) exerts anti-oxidative and anti-inflammatory effects [150,151].

### Alpha-lipoic acid

**Figure 3:**
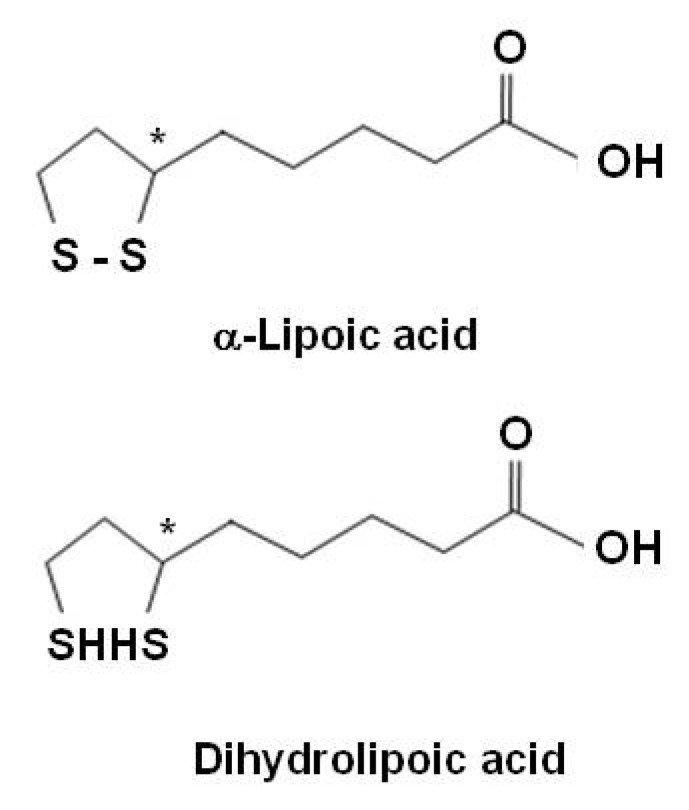
Structures of α-lipoic acid and dihydrolipoic acid.

α-Lipoic acid (LA), an organosulfur compound derived from octanoic acid, is a natural compound also referred as thiothic acid. LA is readily absorbed from the diet and is converted rapidly to its reduced dithiol form, dihydrolipoic acid (DHLA) (Figure 3). Both LA and DHLA are powerful antioxidants [153]. Most LA in food is derived from lipoamide-containing enzymes and is bound to the amino acid, lysine (lipollysine). Plant sources that are rich in lipoyllysine include spinach, broccoli, and tomatoes. LA is a ''non-vitamin" nutrient that is essential to life. It is not classified as a vitamin because it is produced in the body. It is generally involved in oxidative decarboxylations of keto acids and is presented as a growth factor for some organisms. Although LA is involved in cellular energy production, its chief role as a dietary supplement may be as a powerful antioxidant. Unlike other antioxidants, LA is both fat and water-soluble and is easily absorbed and transported across cell membranes. LA directly quenches reactive oxygen species, regenerates/recycles endogenous and exogenous antioxidants such as vitamins C and E and GSH, chelates redox metals including Cu(II) and Fe(II), repairs of oxidised proteins, and modulates the activity of transcription factors such as NF-κB [56,153]. LA has the ability to regenerate other antioxidants like vitamin C, vitamin E, and GSH for further use after they have eradicated free radicals [56].

LA has been used clinically for treating oxidant-induced diseases, such as ischemia-reperfusion injury, cardiovascular disease including atherosclerosis, HIV infection, and diabetic neuropathy [154,155,156,157]. LA also possesses radio-protective properties and furthermore minimizes the pathological consequences of cigarette smoke [158]. In 1986, when Chernobyl nuclear reactor disaster was occurred, LA was found to markedly reduce the ill effects of radiation on liver and kidney function. In addition, LA is very effective in the treatment of heavy metal poisoning and *Aminata* mushroom poisoning [154].

In allergic airway inflammation, LA has been shown to reduce significantly serum IgE concentration, attenuate Th2 cytokines, IL-4, IL-5, IL-13, and IL-18, and reduce NF-κB- activation by decreasing intracellular ROS levels in a murine model of asthma [159]. In addition, LA also suppresses HIF-1α activation linked to VEGF expression that is a critical player in asthma pathogenesis [160]. These reports indicate that LA acts as a multi-functional molecule in airway inflammatory disorders; *i.e.*, a potent anti-oxidant and a modulator of gene expression leading to evoke inflammatory cascades and/or plasma exudation. Until now, there is no clinical study with LA on asthmatic patients, thus, based on these positive data of LA on protective effects for the allergic airway diseases in animal models, we expect well-designed and large scaled clinical trials with LA for asthma to be performed in near future.

### Selenium

Since Schwarz and Foltz established selenium as an essential trace element in the diet for prevention of diseases in 1957, it is well known as a potent nutritional antioxidant [161]. Selenium is derived from both vegetable and animal products, particularly seafood, liver, and cereals. As a member of the sulfur family of elements, selenium shares several chemical properties with sulfur, including valence states and the ability to form covalent bonds with carbon [161]. Selenium is unique among antioxidants in that it exerts its biological effects through direct incorporation into proteins (selenoproteins) as the amino acid selenocysteine. Some selenoproteins that have been characterized as important antioxidant enzymes include GPX-1, GPX-4, thioredoxin reductase-1 and thioredoxin reductase-2, and selenoprotein P [162,163,164]. The selenium-dependent enzyme, GPX recycles glutathione, reducing lipid peroxidation by catalyzing the reduction of peroxides, including hydrogen peroxide [165]. Selenium is also involved in a process of innate and adaptive immune responses [166,167].

Moreover, selenium stabilizes activated platelets which play an important physiological role in allergic processes and immunological mechanisms *i.e*., selenium blocks the allergic inflammation [168]. In asthma, platelets participate by acting as inflammatory cells, by releasing mediators, spasmogens and/or by interacting with other inflammatory cell types [168]. Selenium affects the expression of endothelial cell adhesion molecules, E-selectin, P-selectin, ICAM-1, VCAM-1, and ELAM-1, which are crucial in the inflammatory process for recruitment of inflammatory cells into the target tissue [169,170,171].

Despite of these positive data on selenium use in asthmatics, there are still some conflicting findings of selenium supplementation in animal and human studies for asthma prevalence or severity of asthma, and thus the issue regarding selenium is not conclusive [172,173,174,175].

## Antioxidative Diet

Diet and nutrition may affect the onset and course of chronic inflammatory airway diseases. Serum lycopene and vitamin A concentrations have been found to be significantly lower in asthmatics than in those without asthma [132,133]. In contrast, vitamin E intake is generally unrelated to asthma status but the level of vitamin E in serum is significantly lower in severe asthmatics than in mild asthmatics [133]. Among children, consumption of fresh fruits, particularly fruits high in vitamin C, has been related to a lower prevalence of asthma symptoms and improved lung functions [132,133]. Dietary supplementation or adequate intake of lycopene, vitamin A, flavonoids, and vitamin C rich foods may be beneficial in asthmatic subjects. As for the prevention of the airway disease, many foods may affect development of COPD [132]. Food rich in vitamin C and E may play an especially important role in the prevention and treatment of bronchial asthma.

In fact, studies on lung function decrement and bronchial asthma in adults suggest that daily intake of vitamin C at levels exceeding the current recommended dietary allowance (60 mg/day among nonsmokers and 100 mg/day among smokers) may have a protective effect [132]. In one study, an increase of 40 mg/day in vitamin C intake led to an approximate 20 mL increase in FEV_1_ [132]. An epidemiological study on the impact of dietary selenium and carotenoids on major causes of mortality and morbidity (including asthma) in elderly women showed a lower risk of mortality in subjects with higher serum selenium and serum total carotenoids levels [175].

## Volatile Compounds

### Phytoncides

Phytoncides are natural volatile compounds emitted by trees and plants as a protective mechanism against the harmful insects and animals or microorganism. The major ingredients of phytoncide are highly volatile terpenoids such as α-pinene, carene, and myrcene [176]. Terpenoids are frequently found in essential oils from plants. Monoterpenoids have been isolated from the oils of many higher plants and are valuable in the perfumery and flavor industries. They are also found in nature as insect pheromones and defense secretions and in many marine organisms, where they are usually halogenated. There are four different types of phytoncide solutions introduced by Japanese researchers; chemical components released from trees (A-type), highly bactericidal plants (AB-type), flowering grass (D-type), and non-allergic plants (G-type) [176]. Inhalation of phytoncides is reported as forest bathing and aromatherapy (relaxing effects) [177,178] as well as antibacterial effects [179,180]. Chemical and pharmacological studies have shown that some species produce active principles that exert anti-gastropathic, anti-inflammatory, anti-oxidant, and radical scavenging activity [181,182,183]. It has been reported that physiological effects of phytoncides contribute to the improvement of various disorders including accelerated aging, allergies, multiple sclerosis, and Parkinson disease [176]. 

In addition, phytoncide is reported to enhance the activity of human NK cells [184]. It has also been introduced that fragrance from trees has a regulatory effect on immune function in humans and a restorative effect on the stress-induced immune suppression in mice [185,186].

There is no report about the roles of phytoncide in asthma. However, we have found that administration of phytoncide solution reduces asthmatic phenotypes of an animal model of asthma, restoring GSH level (unpublished data). To define the exact mechanism of phytoncide in asthma, further study is required. In future, we expect that volatile compounds like phytoncide are applicable to the patients with asthma as an additional therapeutic approach.

## Conclusions and Closing Remarks

Asthma is characterized by ongoing inflammation and accompanied by increased oxidative stress and subsequent lung injury. ROS generation through endogenous or exogenous pathway is critical to asthmatic inflammatory responses. Though endogenous antioxidant mechanisms are present to counteract the ROS-mediated inflammatory responses, two opposing mechanisms lose their balance in an inflammatory state. Modulation of these events by enhancing antioxidant levels offers unique opportunities for therapeutic strategies for disease prevention, blocking inflammation or inhibition of airway remodeling. Clearly, the concentrations used in *in vitro* studies (1–100 μM) are at physiologically achievable *in vivo*. Though many animal studies have been revealed possible therapeutic effect of natural bioactive compounds, the available literature regarding dietary manipulation as asthma therapy is largely unconvincing currently. However, there have been remarkable advances continuously in cellular and molecular mechanisms affected by these compounds. Beyond its anti-oxidative activities, plant antioxidants have been demonstrated their anti-inflammatory effects through regulation of various inflammatory cells and mediators, vascular protective effects, and controlling roles in a wide range of signaling pathways. Natural biological compounds could be used alone or in association with other available anti-inflammatory drugs, allowing a reduction in costs and/or side effects. The question remains of whether these data are relevant enough for human disease outcomes, where exposure to natural biological compounds is chronic and at relatively low concentrations, depending on bioavailability and metabolism. The complexity of each reaction and the vast differences in physiologic influences make the clinical research difficult in regard to clinical studies using antioxidant and biologic therapies. Therefore, future studies must include various compounding factors including sufficient dose adjusted, and search for more effective, powerful natural biological compounds should be continued. Based on those studies, the long-term supplementation, large population-based randomized controlled trials with placebo are needed in order to clarify the role and effects of antioxidants in the clinical settings.
